# Multidisciplinary Rehabilitation for People with Parkinson's Disease: A Systematic Review and Meta-Analysis

**DOI:** 10.1155/2022/2355781

**Published:** 2022-02-28

**Authors:** Abubeker Alebachew Seid, Ertugrul Demirdel, Setognal Birara Aychiluhm, Ahmed Adem Mohammed

**Affiliations:** ^1^Department of Nursing, College of Medicine and Health Science, Samara University, Samara, Ethiopia; ^2^Department Physiotherapy and Rehabilitation, Faculty of Health Sciences, Ankara Yildirim Beyazit University, Ankara, Turkey; ^3^Department of Public Health, College of Medicine and Health Science, Samara University, Samara, Ethiopia

## Abstract

**Introduction:**

Guidelines endorse to implement an integrated and multidisciplinary team approach in the management of people with Parkinson's disease (PD). However, there is no net and clear finding that shows the supremacy of multidisciplinary team interventions over conventional interventions for people with PD. Therefore, we perform a systematic review and meta-analysis to determine the supremacy of multidisciplinary interventions for people with PD.

**Methods:**

A systematic review and meta-analysis of randomized controlled trials were conducted. PubMed, Physiotherapy Evidence Database, Cochrane Library, and Google Scholar were searched from inception until May 2021. Randomized controlled trials comparing multidisciplinary intervention with conventional physiotherapy were included. The outcome measures were gait balance, disability status, quality of life, and depression level. The PEDro scale was used to systematically appraise methodological quality. Two reviewers screened, extracted, and performed a quality assessment of included studies independently. Review Manager V.5.4 (Cochrane Collaboration) software was used for statistical analysis. Heterogeneity was analyzed using I^2^ statistics, and a standardized mean difference with 95% CI and *P*value was used to calculate the treatment effect for outcome variables.

**Results:**

A total of 6 studies with 1260 participants were included. The average PEDro methodological quality score was 6.67. No statistically significant difference between multidisciplinary and conventional rehabilitation on functional capacity (SMD: 0.69; 95% CI: −0.13, 1.51; *P*=0.10), disability status (SMD: 0.65; 95% CI: −0.16, 1.46; *P*=0.11), and quality of life (SMD: 0.28; 95% CI: −0.31, 0.59; *P*=0.08) was found. However, there is a statistically significant improvement in caregivers' anxiety levels in the multidisciplinary group (SMD: 0.39; 95% CI 0.06, 1.73; *P*=0.02).

**Conclusion:**

This systematic review and meta-analysis show no significant difference between multidisciplinary and conventional rehabilitation on functionality, disability, and quality of life. Caregivers' anxiety levels show improvement following multidisciplinary interventions. However, large-scale studies with long-term follow-up were required for concrete and clinical recommendations.

## 1. Introduction

Parkinson's disease (PD) is the second most common neurodegenerative disorder next to Alzheimer's disease with approximately 1–2% of the population over 65 years of age suffering from PD [1]. According to the World Health Organization (WHO), 6.1 million individuals have PD globally and it is expected that the trend will continue in the next 30 years having approximately more than 12 million individuals suffering from PD [2, 3]. PD is a progressive disorder characterized by motor and nonmotor symptoms such as rigidity, bradykinesia, resting tremor, autonomic and cognitive dysfunctions, sleep disorders, and sensory disturbances. The combination of these symptoms reduces patients' quality of life (QoL) [3, 4].

There is no known cure for the disease. So, treatments seek to manage symptoms rather than prevent or slow the progression of the disease. Treatments can vary from drugs, surgeries, therapy, or a combination of different treatments. Treatment should also focus on both motor and nonmotor symptoms of the disease. There has been evidence for the inclusion of rehabilitation therapies as an adjuvant to pharmacological and neurosurgical treatment and a call for the move towards multidisciplinary management of this multidimensional condition [1, 3, 5].

Rehabilitation programs that combine both cognitive and physical training like exergames (a portmanteau of exercise and games, aiming to combine the motivational aspects of playing with the physical benefits of exercise) were considered to be feasible, safe, and at least as effective as traditional PD rehabilitation and showed improvement in motor functioning [6]. A broad range of motor and nonmotor symptoms and the need for an individualized treatment approach are best achieved by a multidisciplinary team. This multidisciplinary team might include physical rehabilitation, psychological support, occupational therapy, speech, language, swallowing therapy, and nutrition [2]. A meta-analysis of the current physiotherapy treatment modalities used for PD patients found that conventional physiotherapy, dance, martial arts, Nordic walking, balance, and gait training improved PD motor symptoms and health-related QoL [1, 7].

A study aimed to investigate anxiety, depression, and quality of life in PD patients following multidisciplinary rehabilitation recommended that multidisciplinary rehabilitation improved functional status, mood, motor abilities, autonomy in the activities of daily life, perception of quality of life, cognitive performance, and speech skills in PD patients [8]. Another study that investigated the effectiveness of inpatient multidisciplinary rehabilitation in 68 subjects found that a combination of physical therapy, occupational therapy, and speech therapy for a total of 3 hours per day, 5 to 7 days per week significantly improved all outcome measures [9].

Previous studies have highlighted that integrating different health care professionals into a multidisciplinary care team is needed to tackle the complexity and burden of PD. Even though there is evidence regarding this problem, there is inconsistent in the findings of the previous studies. Therefore, this systematic review and meta-analysis of randomized controlled trials aimed to analyze the supremacy of multidisciplinary rehabilitation over conventional rehabilitation in improving gait, functional mobility, balance outcomes, fall data, disability, and patient-rated quality of life in people with PD.

## 2. Methods

### 2.1. Search Strategy

**Figure 1:**
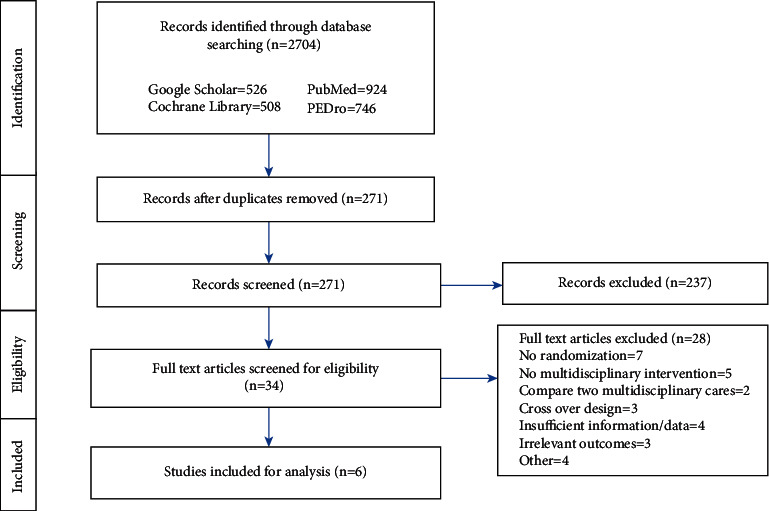
PRISMA flowchart describing the search strategy.

This study was conducted and reported based on the Preferred Reporting Items for Systematic Reviews and Meta-Analyses (PRISMA) guidelines for systematic reviews of randomized controlled trials [10]. A systematic literature search in the PubMed, Physiotherapy Evidence Database, Cochrane Library, and Google Scholar databases were conducted. Articles published from inception to May 2021 were included. Multiple combinations of the following search terms were used: “Parkinson's disease,” “PD,” “physiotherapy,” “multidisciplinary,” “biopsychosocial,” “conventional rehabilitation,” “randomized controlled trial,” “controlled clinical trial,” and “rehabilitation.” A flowchart describing the literature selection steps is shown in Figure 1.

### 2.2. Inclusion Criteria

Full text published randomized controlled trials in English and trials that compare multidisciplinary interventions with conventional physiotherapy interventions in patients with PD were included. Trials were excluded if they did not include at least one of the outcomes of interest measures used. Reviewers (AAS, ED, SBA, and AAM) independently assessed the titles and abstracts according to the inclusion criteria established earlier. Multidisciplinary rehabilitation was defined as rehabilitation aligned with the biopsychosocial model that involves conventional rehabilitation plus a program of any intensity and approach that considers a psychological component or a social/work targeted component. Conventional rehabilitation was defined as interventions used by physiotherapists to manage people with PD, such as exercise, manual therapy, electrotherapy, and other routinely used physiotherapy techniques.

### 2.3. Outcome Measures

The outcomes of interest included gait outcomes (such as the two or six min walk test), functional mobility and balance outcomes (such as the timed up and go test and functional reach test), falls data (such as the number of falls and falls efficacy scale), clinician-rated disability scales (such as the unified Parkinson's disease rating scale-UPDRS) [11], patient-rated quality of life (such as Parkinson's disease questionnaire 39-PDQ-39) [12], anxiety, depression, dopamine use, and other outcomes where data are available.

### 2.4. Data Extraction

Reviewers (AAS, ED, SBA, and AAM) independently assessed the eligible papers and abstracts for study details and outcome data. One reviewer (AAS) extracted data into spreadsheets, and another reviewer (ED) checked for accuracy. Final inclusion was based on consensus between the reviewers. The following information was extracted from the studies: authors, year of publication, randomized comparison, number of participants, eligibility criteria, intervention schedule (including type, duration, and number of sessions), outcome measures, and results.

### 2.5. Methodological Quality

The methodological quality of the included studies was evaluated by using the Physiotherapy Evidence Database (PEDro) scale [13]. When the criteria of each category are met, a point is given, except for criterion number 1, which is not used for the calculation of the total score of the scale. Accordingly, the possible score on the scale ranges from 0 to 10, with a higher score indicating a higher quality in the methods used in the study. A study with a score of 6 or more is considered as evidence level 1 (6–8: good; 9–10: excellent), and a study with a score of 5 or less is considered as evidence level 2 (4–5: fair; <4: poor) [14]. Studies with scores of 6 and above were considered for inclusion. First, one reviewer assessed the methodological quality of each included study, and then a second reviewer checked it independently from the first reviewer. The reviewers agreed on the final quality score of all the included studies.

### 2.6. Statistical Analysis

To perform the analysis, we used Review Manager (RevMan Version 5.4, Copenhagen), the Cochrane Collaboration's software. We used standardized mean difference (SMD) along with the 95% confidence interval and significance level set to *P* < 0.05. The *I*^2^ statistics test was used to test heterogeneity, and a fixed-effect model or random-effects model was selected based on the result. For scales indicating improvement by decreasing (such as UPDRS and PDQ-39), mean values were adjusted by multiplication with −1. An effect size of less than 0.2 was considered as a small effect, 0.2 to 0.5 as a moderate effect, and more than 0.8 as a large effect [7].

## 3. Results

### 3.1. Study Characteristics

We identified 2704 potentially eligible studies from the four databases searched. We excluded irrelevant studies and duplicates, and 34 full-text studies were assessed for details. From these, 6 full-text studies with a total sample size of 1260 people fulfilled the inclusion criteria and were used for the analysis. The reasons for exclusion were inadequate randomization, non-multidisciplinary interventions, cross-over design, insufficient information, irrelevant outcomes, and other factors. The sample size included in the studies ranged from 43 to 762, and the inclusion and exclusion criteria were clearly stated in all studies.

In all studies, a multidisciplinary team was involved including an occupational therapist, psychological therapist, movement disorder specialist, social worker, and others. Five interventions used inpatients and/or health facility-based treatment while one study focused on home-based treatment. The duration of the intervention ranges from 4 weeks to 8 months. There was substantial heterogeneity in the details of intervention and the outcomes of interests measured. The characteristics of all included studies are described in Table 1.

### 3.2. Outcome Measures

The most common outcome measures reported in the studies were gait outcomes, functional mobility, balance and fall outcomes, motor symptoms, and patient-rated quality of life. Physical performance outcomes were assessed using Nottingham Extended Activities of Daily Living (NEADL) [15], Timed Up and Go Test (TUG) and Berg Balance Scale (BBS) [4], BBS and functional independence measure (FIM) [16], and Canadian Occupational Performance Measure (COMP) [17] as reported in the trials. Motor and cognitive outcomes were evaluated using the Unified Parkinson's Disease Rating Scale part III (UPRS-III) reported in 4 trials [15, 17–19]. UPRS-III and UPDRS reported total in 2 trials [18, 19]. The UPDRS total alone was reported only in one trial [4].

In assessing psychological symptoms such as depression and anxiety, the Montgomery Asberg Depression Scale (MADRS) and Scales for Outcomes in Parkinson's Disease Psychosocial (SCOPA) [18], Geriatric Depression Scale (GDS) [19], State-Trait Anxiety Inventory (STAI), and Beck Depression Inventory (BDI) [4] were reported. In the assessment of participants' quality of life, 4 studies used PDQ-39 [4, 15, 16, 18] and one trial used EQ-5D and SF-12 in addition to PDQ-39 [15]. One trial used PDQL [19] for the assessment of the patient rated the quality of life and no quality-of-life outcome measurement tool was mentioned in one trial [17].

Four trials reported pretest daily use of dopaminergic drugs, but post-test data were missing [15, 16, 18, 19]. In three trials [4, 16, 18], multidisciplinary rehabilitation was found to be superior and effective in PD treatment, while two trials found no evidence to small effect in favor of multidisciplinary intervention [15, 17], and one other trial found no difference in both multidisciplinary and control groups [19] (Table 1).

## 4. Methodological Quality

The PEDro methodological quality assessment of the included 6 studies showed that all studies had good (average 6.67) quality scores. Three trials scored 6, two trials scored 7, and one trial scored 8. Only one trial could succeed to blind all the participants, therapist, and assessor, and the other two trials described whether they used an intention to treat analysis or not. The methodological quality scores of the included trials are shown in Table 2.

### 4.1. Functional Capacity

**Figure 2:**
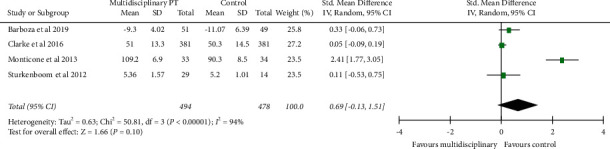
Forest plot multidisciplinary physiotherapy with the control group: functional mobility.

As shown in the meta-analysis forest plot (Figure 2), four studies with 972 patients' functional status/capacity outcomes showed statistically no significant difference between multidisciplinary rehabilitation and conventional rehabilitation (SMD: 0.69; 95%CI: −0.13, 1.51; *P*=0.10).

### 4.2. Balance and Fall Data

Two studies reported data for balance and fall records. However, data for the control group was missed in one study. The corresponding author was contacted for the missing data, but we received no response. Results of those studies showed that both balance and fall outcomes improved after multidisciplinary rehabilitation.

### 4.3. Disability Status

**Figure 3:**
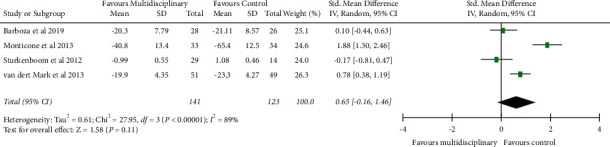
Forest plot multidisciplinary physiotherapy with the control group: disability status.

From the data of four studies (243 people), the clinician-rated disability status (UPDRS motor score) was not significantly improved (Figure 3) with multidisciplinary rehabilitation compared with conventional rehabilitation (SMD: 0.65; 95% CI: −0.16, 1.46; *P*=0.11).

### 4.4. Quality of Life

**Figure 4:**
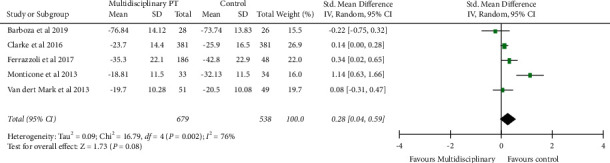
Forest plot multidisciplinary physiotherapy with the control group: quality of life.

Five studies used appropriate measures of quality of life (PDQ-39) in patients with PD. Multidisciplinary physiotherapy has a moderate but statistically insignificant effect (Figure 4) on quality of life compared to conventional care (SMD: 0.28; 95% CI: −0.31, 0.59; *P*=0.08).

### 4.5. Anxiety and Depression

**Figure 5:**
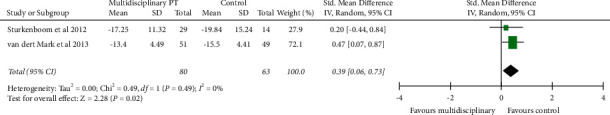
Forest plot multidisciplinary physiotherapy with the control group: anxiety (caregiver).

Only two studies reported data on caregiver anxiety (strain) level, and the meta-analysis result showed that multidisciplinary rehabilitation has a moderate and significant effect (Figure 5) on caregiver strain over conventional physiotherapy with an average of SMD 0.39 (95% CI: 0.06, 1.73; *P*=0.02).

### 4.6. Daily Dopamine Use

Five studies have reported data on the daily use of dopaminergic drugs (LED mg) in the preintervention phase. But postintervention data was missing in four studies; consequently, we were unable to perform the meta-analysis on daily dopamine use.

## 5. Discussion

Our study aimed to determine the superiority of multidisciplinary approach rehabilitation over conventional rehabilitation in patients with PD. This review provides evidence on the efficacy of multidisciplinary rehabilitation over conventional rehabilitation in the short-term (mean follow-up three months) treatment of PD. In this study, a moderate quality effect of multidisciplinary rehabilitation was found for functional status, disability, and patient-rated quality of life, but all those outcomes were statistically non-significant. By contrast, only the caregiver's anxiety level showed significant benefit from treatment with multidisciplinary rehabilitation. The reason for these unexpected results might be secondary to the small size and short duration of interventions in many of the included studies [20].

While comparing the effects of multidisciplinary rehabilitation and conventional rehabilitation on functional capacity, the results failed to reach the statistical significance level to show the difference, even though a moderate level of improvement was seen in the multidisciplinary group. Similar to our study, a trial comparing physiotherapy and occupational therapy found no clinically meaningful improvement on functional status and quality of life in immediate and medium-term measurements [15]. Another Cochrane review of physiotherapy vs no intervention in PD showed that physiotherapy produced small benefits in motor function and ADL but no change in QoL [20]. The possible explanation for this insignificant outcome is that each approach used may have a positive impact on functional status and the limited study duration could not show the superiority of the compared interventions. In addition, the multifactorial nature of the disease, the low “dose” of the interventions, and lack of consistency in therapy outcome assessment and intervention might have contributed to this insignificant result.

Our meta-analysis failed to show a statistically significant difference between multidisciplinary rehabilitation and conventional rehabilitation on disability status in patients with PD. These findings are similar to the findings of other studies [17, 21]. One possible reason may be that both treatment approaches have a beneficiary effect and the sample size was not adequate to detect the difference. A study on inpatient enhanced multidisciplinary care (EMC) and multidisciplinary rehabilitation in an 8-week intervention found significant improvements in QoL and other motor and nonmotor symptoms [22]. In this review, no significant improvement was found between multidisciplinary and conventional rehabilitation groups. This may be secondary to the nonsignificant improvements in functional capacity and disability status because these are important factors potentially affecting the QOL in PD. A trial study conducted to assess the effectiveness of the inclusion of mental practice on standard physiotherapy at improving mobility tasks in people with PD found no statistically significant difference in the groups [23]. The overall results can be concluded that rehabilitation approaches for such chronic diseases like PD should focus on a long-term basis rather than the short- and medium-term. Our finding of nonsuperiority in a multidisciplinary team is consistent with the known progress of the disease, or the studies included might have been too small to detect the difference.

Our study had limitations that need to be considered. Firstly, the search was limited to the English language only. Quality articles published in other languages were missed. Secondly, we used the PEDro scale for methodological quality assessment. The scale has some limitations. For example, it focuses on quality of reporting rather than factors that influence the risk of bias (as recommended by PRISMA guidelines and the Cochrane Collaboration) and it does not consider the timing of outcomes or compliance with the intervention, which are highly relevant when reviewing physiotherapy interventions. Thirdly, the sample sizes of the studies included in this review are still small.

## 6. Conclusions

Multidisciplinary rehabilitation showed statistically no significant effects on functional capacity, disability status, and quality of life in PD patients compared to conventional rehabilitation. In contrast, multidisciplinary rehabilitation showed a statistically significant effect on anxiety (caregivers) symptoms compared to conventional rehabilitation. These results suggest that multidisciplinary rehabilitation may not show superiority over conventional rehabilitation in patients with PD. However, due to the small number of randomized controlled trials and methodological limitations, we are unable to draw concrete conclusions. Therefore, further studies with better designs and an adequate sample size will be needed.

## Figures and Tables

**Table 1:** Characteristics of the included studies.

Author (yr.)	*N*	Inclusion criteria	Exclusion criteria	Intervention	Duration	Outcome measures	Result
Van der Mark et al. (2013)	IG = 51	Clinical diagnosis of PD, ability to complete the study questionnaires, written informed consent, and presence of a caregiver	Dementia (MMSE <24) and current treatment by a movement disorders specialist	Multidisciplinary team (movement disorders specialist, PD nurses, and social worker)	8 months	PDQ-39, UPDRS part III, UPDRS total, MADRS, SCOPA-PS, CSI, and daily LED (mg)	Credence to a multidisciplinary team approach
	CR = 49			*∗*Control group: care given by a general neurologist			
Barboza et al. (2019)	IG = 28	Idiopathic PD, stages 1.5 to 3 on the modified H&Y scale, older than 50 yrs, and independent for walking	Other neurological, musculoskeletal, and associated disorders, as well as cognitive alterations, that interfere with movement	Motor physiotherapy with cognitive training	4 months	UPDRS (domain II), UPDRS (domain III), UPDRS (total), GDS, MMSE, MoCA, LED (mg), and PDQL	Both treatment approaches were effective for the outcomes
	CG = 26			*∗*Control group: motor PT only			
Clarke et al. (2016)	IG = 381	Idiopathic PD defined by the UK Parkinson disease society brain bank criteria; self- or caregiver reported limitations in ADL	Dementia as locally defined and receipt of PT or OT for PD in the last 12 months	Physiotherapy plus occupational therapy	8 weeks	NEADL scale, PDQ-39, EQ-5D, and SF-12	No evidence to support this intervention
	CG = 381			*∗*Control group: no therapy			
Ferrazzoli et al. (2017)	IG = 186	Idiopathic PD by the UK brain bank criteria, H&Y stages 2–4, and stable pharmacological treatment in the last 6 weeks	Any focal brain lesion (CT or MRI), psychosis, auditory, visual, and/or vestibular dysfunctions and other chronic diseases	Multidisciplinary intensive rehabilitation treatment (MIRT)	4 weeks	PDQ-39, UPDRS total, PDDS, TUG, BBS, STAI, BDI, LED (mg), and neurologic tests	MIRT improve QoL in short-term and long-term period
	CG = 48			*∗*Control group: no rehabilitation			
Monticone et al. (2015)	IG = 33	Idiopathic PD (modified H&Y scale, 2.5–4), a decline in function assessed, older than 50 yrs, duration of more than 10 yrs, and stable drug use for more than 15 days	Dementia and other neurological diseases, systemic illness, psychiatric deficits, invasive drug treatments, and surgical interventions for PD	Multidisciplinary rehabilitative (MR) care-motor training, cognitive training, and ergonomic education	8 weeks	UPDRS part III, BBS, FIM and PDQ-39	MR improves patient conditions
	CG = 34			*∗*Control group: general physiotherapy			
Sturkenboom (2012)	IG = 29	Idiopathic PD lived at home, reported difficulties in daily activities, had a nonprofessional caregiver who could assist at least twice a week	Use of occupational therapy in the last 12 months, disabling comorbidity, inability to complete questionnaires, participation in another intervention trial	Home-based occupational therapy according to the Dutch guidelines of occupational therapy	10 weeks	UPDRS III, CIRS-G, MMSE, COPM, AMPS, and ZBI	Negligible to small effects in favor of the intervention group
	CG = 14			*∗*Control group: no occupational therapy			

IG: intervention group, CG: control group, PD: Parkinson's disease, H &Y scale: Hoehn and Yahr scale, ADL: activity of daily living, MIRT: multidisciplinary intensive rehabilitation treatment, MMSE: mini-mental state examination, PDQ-39: Parkinson's Disease Questionnaire, UPDRS: Unified Parkinson Disease Rating Scale, MADS: Montgomery Asberg Depression Scale, SCOPA-PS: Scales for Outcomes in Parkinson's Disease Psychosocial Index, CSI: Caregiver Strain Index, LED: levodopa equivalent dose, GDS: Geriatric Depression Scale, MoCA: Montreal Cognitive Assessment, PDQL: Parkinson's Disease Quality of Life Questionnaire, NEADL: Nottingham Extended Activities of Daily Living, EQ-5D: EuroQol-5D, SF-12: short form 12, PDDS: Parkinson's Disease Disability Scale, TUG: Timed Up and Go Test, BBS: Berg Balance Scale, STAI: State-Trait Anxiety Inventory, BDI: Beck Depression Inventory, FIM: functional independence measure, CIRS-G: Cumulative Illness Rating Scale-Geriatrics, COPM: Canadian Occupational Performance Measure, AMPS: Assessment of Motor and Process Skills, and ZBI: Zarit Burden Inventory.

**Table 2:** The PEDro methodological quality score for studies included (*Y* = yes and *N* = no).

Study author/s (year)	1	2	3	4	5	6	7	8	9	10	11	Total score	Methodological quality
Van der Mark et al. (2013)	Y	Y	N	Y	Y	Y	Y	N	N	Y	Y	7	Good
Barboza et al. (2019)	Y	Y	Y	Y	N	Y	Y	N	Y	Y	Y	8	Good
Ferrazzoli et al. (2017)	Y	Y	Y	Y	N	N	Y	Y	N	Y	Y	7	Good
Clarke et al. (2016)	Y	Y	N	Y	N	N	N	Y	Y	Y	Y	6	Good
Monticone et al. (2015)	Y	Y	N	Y	N	N	Y	Y	N	Y	Y	6	Good
Sturkenboom (2012)	Y	Y	N	Y	N	N	Y	Y	N	Y	Y	6	Good

**1.** Eligibility criteria were specified. **2**. Subjects were randomly allocated to groups. **3**. The allocation was concealed. **4**. The groups were similar at baseline regarding the most important prognostic indicators. **5**. There was a blinding of all subjects. **6**. There was blinding of all the therapists who administered the therapy. **7**. There was blinding of all assessors who measured at least one key outcome. **8**. Measures of at least one key outcome were obtained from more than 85% of the subjects initially allocated to groups. **9**. All subjects for whom outcome measures were available received the treatment or control condition as allocated or, where this was not the case, data for at least one key outcome was analyzed by ‘‘intention to treat.” **10**. The results of between-group statistical comparisons are reported for at least one key outcome. **11**. The study provides both point measures and measures of variability for at least one key outcome.

## Data Availability

The data supporting this systematic review meta-analysis are from previously published studies, which have been cited. The processed data are available from the corresponding author upon reasonable request.

## Supplementary Materials

Supplementary Materials Appendix 1: PRISMA 2020 checklist. .
